# Sodium Bicarbonate Therapy in Patients with Metabolic Acidosis

**DOI:** 10.1155/2014/627673

**Published:** 2014-10-21

**Authors:** María M. Adeva-Andany, Carlos Fernández-Fernández, David Mouriño-Bayolo, Elvira Castro-Quintela, Alberto Domínguez-Montero

**Affiliations:** Nephrology Division, Hospital General Juan Cardona, Avenida Pardo Bazán, s/n, Ferrol, 15406 A Coruña, Spain

## Abstract

Metabolic acidosis occurs when a relative accumulation of plasma anions in excess of cations reduces plasma pH. Replacement of sodium bicarbonate to patients with sodium bicarbonate loss due to diarrhea or renal proximal tubular acidosis is useful, but there is no definite evidence that sodium bicarbonate administration to patients with acute metabolic acidosis, including diabetic ketoacidosis, lactic acidosis, septic shock, intraoperative metabolic acidosis, or cardiac arrest, is beneficial regarding clinical outcomes or mortality rate. Patients with advanced chronic kidney disease usually show metabolic acidosis due to increased unmeasured anions and hyperchloremia. It has been suggested that metabolic acidosis might have a negative impact on progression of kidney dysfunction and that sodium bicarbonate administration might attenuate this effect, but further evaluation is required to validate such a renoprotective strategy. Sodium bicarbonate is the predominant buffer used in dialysis fluids and patients on maintenance dialysis are subjected to a load of sodium bicarbonate during the sessions, suffering a transient metabolic alkalosis of variable severity. Side effects associated with sodium bicarbonate therapy include hypercapnia, hypokalemia, ionized hypocalcemia, and QTc interval prolongation. The potential impact of regular sodium bicarbonate therapy on worsening vascular calcifications in patients with chronic kidney disease has been insufficiently investigated.

## 1. Measurement of Plasma Bicarbonate

The analysis of blood gases includes three parameters related to the carbon dioxide (CO_2_) content of blood: total concentration of carbon dioxide in blood (tCO_2_), plasma partial pressure of carbon dioxide (pCO_2_), and plasma bicarbonate (HCO_3_^−^) concentration [1, 2].

The plasma pCO_2_ is measured by blood gases analyzers and indicates the pressure exerted by the small portion (approximately 5%) of total carbon dioxide dissolved in the aqueous phase of plasma. The concentration of plasma bicarbonate is usually estimated from measured pH and pCO_2_ values when blood gas analyzers are utilized. The tCO_2_ is chemically measured by laboratory analyzers and reflects the total amount of carbon dioxide present in blood, which primarily corresponds to the sum of bicarbonate and dissolved carbon dioxide or pCO_2_ [1, 3].

The relationship between pH and pCO_2_ used to calculate plasma bicarbonate concentration is described by the Henderson-Hasselbalch equation derived from the application of the law of mass action to the hydration and dissociation reactions of carbonic acid (H_2_CO_3_) in plasma:

(1) $$\begin{array}{c} {\text{CO}}_{2}+{\text{H}}_{2}\text{O}⟷{\text{H}}_{2}{\text{CO}}_{3}\\ {\text{H}}_{2}{\text{CO}}_{3}⟷{\text{H}}^{+}+{{\text{HCO}}_{3}}^{-} \end{array}$$

The equilibrium constant for the first reaction (*K*_1_) is

(2) $$\begin{array}{c} K_{1}=\frac{\left[{\text{H}}_{2}\text{C}{\text{O}}_{3}\right]}{\left[\text{C}{\text{O}}_{2}\right]\left[{\text{H}}_{2}\text{O}\right]}. \end{array}$$

The equilibrium constant for the second reaction (*K*_2_) is

(3) $$\begin{array}{c} K_{2}=\frac{\left[{\text{H}}^{+}\right]\left[{{\text{HCO}}_{3}}^{-}\right]}{\left[{\text{H}}_{2}{\text{CO}}_{3}\right]}. \end{array}$$

The Henderson-Hasselbalch equation used for the calculation of plasma bicarbonate is

(4) $$\begin{array}{c} \text{pH}=\text{p}K_{a}+\log\frac{\left[{{\text{HCO}}_{3}}^{-}\right]}{\alpha \times \text{p}{\text{CO}}_{2}}. \end{array}$$

The p*K*_*a*_ value for this equation is obtained from a combined equilibrium constant including the values of *K*_1_ and *K*_2_. The p*K*_*a*_ of this new combined constant (6.1) is used in the calculation of plasma bicarbonate concentration. *α* is the solubility coefficient for carbon dioxide gas (equal to 0.0306 for plasma at 37°C).

Substituting these values,

(5) $$\begin{array}{c} \text{pH}=6.1+\log\frac{\left[{{\text{HCO}}_{3}}^{-}\right]}{0.03\times \text{p}{\text{CO}}_{2}}. \end{array}$$

**Figure 1:**
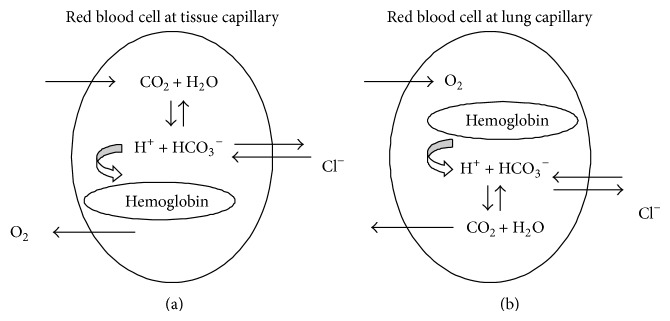
Carbonic anhydrase reaction.

Blood gases analyzers use this formula to estimate plasma bicarbonate concentration from known pH and pCO_2_ values. However, the application of this equation to the calculation of the bicarbonate concentration in human plasma may be misleading, as the hydration of carbon dioxide in vivo requires the action of isoenzymes of carbonic anhydrase which either are anchored to the plasma membrane of red blood cells or lie inside the erythrocytes. Activity of carbonic anhydrase isoenzymes has not been reported in human plasma in sufficient amount to drive significantly the hydration of carbon dioxide on this location. In addition, isoforms of carbonic anhydrase catalyze the reversible hydration of carbon dioxide into bicarbonate with no intermediate formation of carbonic acid (Figure 1) [4]. Therefore, the use of a combined p*K*_*a*_ may not be appropriate, as there is no carbonic acid formation in vivo. For these reasons, the application of the Henderson-Hasselbalch equation to the calculation of the plasma bicarbonate concentration is not straightforward and the physiological meaning of the plasma bicarbonate value estimated from the application of this equation to human plasma remains uncertain.

The tCO_2_ in blood determined by laboratory analyzers and the calculated plasma bicarbonate concentration from point-of-care blood gases analyzers are usually considered equivalent and the tCO_2_ is generally measured as a surrogate for plasma bicarbonate level, although some studies have found poor agreement between the two parameters [3, 5].

## 2. Sodium Bicarbonate Therapy in Metabolic Acidosis

Metabolic acidosis is usually associated with a reduction in plasma pH, although serum concentration of hydrogen ions may be near normal when a mixed acid-base disorder is present. For instance, the coexistence of vomiting-induced metabolic alkalosis may contribute to the rise in plasma pH in patients with metabolic acidosis. Common causes of metabolic acidosis include diabetic ketoacidosis (DKA), lactic acidosis, and hyperchloremic acidosis due to diarrhea or renal tubular acidosis. Excess net dietary acid load in the presence of chronic kidney dysfunction induces metabolic acidosis with elevation of chloride and unmeasured anions. In acute conditions, such as DKA, lactic acidosis, and septic shock, the magnitude of the fall in plasma pH usually reflects the severity of the causative illness. Evidence that significant harmful effects are derived from metabolic acidosis by itself has not been provided in human beings [6–10] and therefore the successful management of metabolic acidosis requires the therapy of the underlying causative disorder [11]. Replacement of sodium bicarbonate is beneficial in disorders associated with loss of sodium bicarbonate, such as diarrhea and renal tubular acidosis, but symptomatic therapy with sodium bicarbonate to correct metabolic acidosis per se in other settings has not been demonstrated to ameliorate clinical outcomes or mortality (Table 1) [8, 10, 12–14]. Further, sodium bicarbonate supplementation fails to raise plasma pH or increases it only slightly in some patients affected with malignancy-associated lactic acidosis, while control of the underlying malignancy brings plasma pH to normal [15–22].

### 2.1. Sodium Bicarbonate Therapy in Patients with Diabetic Ketoacidosis

Both retrospective and prospective studies have consistently documented that sodium bicarbonate therapy does not improve metabolic responses, biochemical parameters, acid-base balance normalization, or clinical outcomes among patients with DKA, either children or adults. The rate of decline of blood glucose, the mean time to achieve an arterial pH ≥ 7.30, and the recovery rates of plasma bicarbonate level and pH are similar among DKA patients with or without sodium bicarbonate infusion [23–32]. The lack of benefit from sodium bicarbonate therapy in the management of DKA has been also confirmed in patients with severe DKA, with plasma pH values between 6.9 and 7.1 and less than 6.9 [24, 27, 30, 33, 34].

### 2.2. Sodium Bicarbonate Therapy in Patients with Lactic Acidosis

No benefit from sodium bicarbonate therapy has been found in the management of lactic acidosis regarding clinical outcomes or mortality [35]. High doses of sodium bicarbonate have failed to improve lactic acidosis induced by malignancy while the acidosis subsides after chemotherapy [16–19]. Other causes of lactic acidosis including sepsis and phenphormin-induced lactic acidosis are also resistant to bicarbonate therapy [20, 36]. Two prospective randomized crossover trials enrolling critically ill patients with metabolic acidosis and elevated blood lactate concentration have not found benefit from the use of sodium bicarbonate compared to sodium chloride on hemodynamic responses, even in very acidemic participants [21, 22]. A recent retrospective single-center trial has evaluated the effect of sodium bicarbonate on mortality rate among patients with lactic acidosis, concluding that sodium bicarbonate administration is independently associated with higher mortality [37].

### 2.3. Sodium Bicarbonate Therapy in Patients with Septic Shock

In patients diagnosed with septic shock, sodium bicarbonate therapy has not been associated with improvement of hemodynamic variables or mortality rate in retrospective [38] and prospective [39] studies. Accordingly, the 2008 update of the Surviving Sepsis Campaign guidelines recommends against the use of sodium bicarbonate in patients with hypoperfusion-induced lactic acidosis and pH ≥ 7.15 [40].

### 2.4. Sodium Bicarbonate Therapy in Patients with Intraoperative Metabolic Acidosis

A negative impact on mortality has been reported following the use of sodium bicarbonate in a retrospective cohort study of severely acidotic (arterial pH ≤ 7.10) trauma patients who underwent emergency surgery [14]. No benefit from bicarbonate therapy has been found either in a small prospective randomized trial of patients who developed intraoperative mild metabolic acidosis in the absence of hypoxemia [41].

### 2.5. Sodium Bicarbonate Therapy in Patients with Cardiac Arrest

Sodium bicarbonate therapy has long been removed from guidelines for advanced cardiac life support, as a review of the medical literature shows no beneficial effect of sodium bicarbonate on survival rates in this setting [13].

### 2.6. Sodium Bicarbonate Therapy in Patients with Acute Kidney Disease

No randomized controlled trials have investigated the effect of sodium bicarbonate therapy in acute kidney injury, excluding studies that evaluated the use of sodium bicarbonate for acute kidney injury prevention [42].

### 2.7. Sodium Bicarbonate Therapy in Patients with Chronic Kidney Disease

Long-lasting therapy with sodium bicarbonate is extensively used for management of metabolic acidosis associated with chronic kidney disease (CKD), as current guidelines suggest sodium bicarbonate supplementation to maintain serum bicarbonate ≥ 22 mmol/L (mM) (level of evidence 2B) [43]. In addition, administration of sodium bicarbonate to patients with CKD has been suggested in recent years as a renoprotective approach to delay the deterioration of kidney function. However, in Cochrane [44] or systematic [45] reviews of the medical literature, there is no conclusive evidence to support alkali therapy with sodium bicarbonate in patients with CKD.

#### 2.7.1. Prevalence of Metabolic Acidosis Associated with CKD

A significant reduction in serum bicarbonate concentration occurs in advanced CKD, when the glomerular filtration rate (GFR) is approximately ≤ 20 mL/min [46–50]. Among participants of the Third National Health and Nutrition Examination Survey (NHANES), 19% of subjects with GFR 15–29 mL/min/1.73 m^2^ show a serum bicarbonate level < 22 mM [46]. Similarly, other studies report that approximately 75% of patients with estimated GFR (eGFR) 15–60 mL/min show serum bicarbonate concentration ≥ 23 mM and only 5% have serum bicarbonate level ≤ 19 mM [51].

#### 2.7.2. Cause of Metabolic Acidosis Associated with CKD

In patients with CKD, metabolic acidosis is associated with an elevation of serum chloride or unmeasured anions or both. Early in the course of CKD, several studies have documented an increase in serum chloride level whereas the anion gap, calculated from the equation [Na^+^] − ([Cl^−^] + [HCO_3_^−^]), remains normal. In more advanced CKD there is a progressive rise in the serum anion gap while serum chloride usually continues to be elevated [46, 47, 49, 50, 52, 53]. However, the anion gap may be modified by other plasma ions, such as albumin, phosphate, and potassium, which are usually altered in patients with CKD. When the anion gap is calculated taking into account the level of these ions, this parameter begins to be increased at eGFR of 60–89 mL/min/1.73 m^2^, indicating that higher levels of unmeasured anions may be present in patients with mild kidney disease [54]. The concentration of serum phosphate increases steadily throughout the course of CKD. As phosphate is an unmeasured anion, hyperphosphatemia may partially explain the elevated anion gap [47, 50]. Plasma albumin is also an unmeasured anion and therefore hypoalbuminemia, which is frequently present in patients with CKD, decreases the anion gap. Data from participants of the NHANES 1999–2004 show that there is a graded rise in the albumin-adjusted anion gap across eGFR categories beginning with eGFR 45–59 mL/min/1.73 m^2^ [54]. Serum potassium concentration displays a stepwise increase among patients with kidney disease becoming first evident at GFR of 50–60 mL/min [47] while serum sodium concentration in patients with CKD is not different from control groups [50, 52].

The fall in plasma pH present in patients with advanced CKD is due to the inability of the ailing kidney to effectively handle the dietary acid load imposed by excess animal protein and chloride intake. After a meat load, healthy persons maintain acid-base parameters in the normal range, while patients with CKD develop slight metabolic acidosis, indicating that the acid load imposed to the kidney by the meat load exceeds its excretory capacity [55]. In healthy subjects, the intake of animal proteins is associated with an increase in renal plasma flow and GFR, while vegetable proteins do not induce renal vasodilatation or glomerular hyperfiltration. Further, the effect of chronic meat ingestion is abolished by vegetable protein ingestion and a marked reduction in GFR and renal plasma flow is observed during vegetable protein intake [56, 57]. The differential effect of vegetable and animal proteins on kidney hemodynamics is also apparent in diabetic patients, which show lower GFR and renal plasma flow during the consumption of vegetable protein diets compared to animal protein diets [58]. In addition, vegetarian diets reduce the urinary albumin excretion rate in healthy individuals, patients with CKD, and diabetic patients compared with animal protein diets [55, 58].

A cross-sectional evaluation of data from the Dortmund Nutritional and Anthropometric Longitudinally Designed study shows that the ability of the kidney to excrete an acid load (renal net acid excretion capacity) declines with age in healthy adults, such that older persons have lower capacity to excrete an acid load [59]. Accordingly, data from the NHANES 1999–2004 have detected an inverse association between dietary acid load and serum bicarbonate levels among middle-age and elderly participants. However, serum bicarbonate levels among young participants did not differ by dietary acid level, suggesting that younger subjects are able to handle the acid load without altering the acid-base balance [60]. Similar to healthy subjects, dietary protein intake is inversely associated with serum bicarbonate concentration in patients with CKD, indicating that some degree of the plasma acidosis present in these patients is due to excess net dietary acid load. Among participants in the Modification of Diet in Renal Disease (MDRD) study, serum tCO_2_ is inversely related to dietary protein intake in a cross-sectional analysis of baseline data. In the longitudinal analysis, an intentional reduction in protein intake leads to an increase in serum tCO_2_ after adjustment for covariates [61]. Likewise, an inverse association between net endogenous acid production (NEAP) and serum bicarbonate levels has been detected among African American adults with hypertensive CKD in a cross-sectional analysis. Higher NEAP is associated with lower serum bicarbonate concentration in a graded fashion [62]. A similar association has been observed in kidney transplant recipients in which patients with high intake of animal protein and low intake of fruits and vegetables have lower serum bicarbonate and serum pH [63].

#### 2.7.3. Metabolic Acidosis and Mortality Rate in Patients with CKD

In patients with moderate and advanced CKD, the association between serum bicarbonate concentration and all-cause mortality is U-shaped. The lowest mortality rate is seen in patients with serum bicarbonate concentration of 26–29 mM. The highest mortality rate is observed among patients with serum bicarbonate levels of < 22 mM but an increase in mortality is also seen in patients with serum bicarbonate levels of > 29 mM [64].

#### 2.7.4. Relationship between Metabolic Acidosis and Kidney Disease Progression

Results from recent clinical trials might suggest that metabolic acidosis may contribute to progression of kidney dysfunction in patients with CKD, but evidence is inconclusive and additional investigations are needed (Table 2) [65, 66].

A retrospective observational study enrolling 5,422 adults has found an association between low serum bicarbonate concentration and progression of kidney disease. Patients with baseline serum bicarbonate level < 22 mM bear a slightly higher risk of a composite renal outcome defined as either a decrease in the eGFR by 50% or reaching an eGFR < 15 mL/min/1.73 m^2^. However, only 9% of the participants have eGFR less than 60 mL/min/1.73 m^2^ at baseline and those with the lowest eGFR at baseline also had lower serum bicarbonate levels, suggesting that progression may be influenced by the baseline level of kidney function. In addition, the only inclusion criterion in this study was being an adult visiting a medical clinic with two blood samples drawn. Therefore, the estimation of progression of kidney dysfunction is very limited. Being a retrospective observational analysis, causality cannot be inferred [65, 67].

A secondary analysis of the African American Study of Kidney Disease and Hypertension (AASK) trial database including 1,094 patients has suggested that higher serum bicarbonate is associated with reduced hazard of CKD progression. After adjusting for baseline iothalamate clearance and baseline proteinuria, each 1 mM increase in serum bicarbonate is associated with a 4% lower hazard of the clinical composite outcome of death, dialysis, or GFR event (hazard ratio 0.950, 95% confidence interval 0.916–0.985). However, the external validity of this study is limited, as more than 50% of participants in the AASK trial die or develop a doubling of serum creatinine or end-stage renal disease (ESRD) at 10 years of follow-up [68].

The role of serum bicarbonate level as a risk factor for renal outcomes (ESRD or 50% reduction in eGFR) has been evaluated in 3,939 participants with CKD stages 2–4 enrolled in the Chronic Renal Insufficiency Cohort (CRIC) trial, a prospective multicenter cohort study. After adjustment for covariates, the risk of developing a renal end point is 3% lower per 1 mM increase in serum bicarbonate level (hazard ratio 0.97, 95% confidence interval 0.94–0.99) [51].

The association between a dietary pattern resulting in higher NEAP and progression of kidney disease (time to ESRD or doubling of serum creatinine) has been investigated in 632 participants in the AASK trial, but no definite conclusion could be attained. After adjustment for covariates, higher quartiles of NEAP are associated with a faster decline in GFR over follow-up, estimated by the slope of iothalamate GFR. In addition, higher quartiles of NEAP remain associated with higher rate of decline in iothalamate GFR after further adjustment for serum bicarbonate, indicating that the potential association between NEAP and CKD progression is independent of serum bicarbonate. However, in time-to-event analyses over a median of 7.7 years, there is not a statistically significant association between higher NEAP and composite renal events (time to ESRD or doubling of serum creatinine) [69].

The association between serum bicarbonate level and CKD progression (measured as a fall of 25% or more in eGFR or starting dialysis) has been examined in a retrospective study enrolling 113 elderly patients with average eGFR 25.7 mL/min/1.73 m^2^ and average serum bicarbonate concentration 27.4 mEq/L. Baseline data in this study show that patients in the low-bicarbonate group (serum bicarbonate concentration < 23 mM) have markedly lower eGFR (15.1 mL/min/1.73 m^2^ versus 29.1 mL/min/1.73 m^2^) and higher 24-hour urinary protein excretion level (1.25 g versus 0.83 g), compared with participants in the high-bicarbonate group (serum bicarbonate level > 23 mM). Although multivariate regression analysis suggests that lower bicarbonate level is associated with high risk of CKD progression, the start of dialysis may be related to strikingly more advanced kidney disease among patients with lower bicarbonate concentration. In addition, the relatively small sample size and the high number of censored observations indicate that this study may be statistically underpowered to detect independent associations between serum bicarbonate concentration and CKD progression [70].

No association between low bicarbonate concentration and risk of incident kidney dysfunction has been found in two recent retrospective cohort studies [71, 72].

The association between serum bicarbonate level and risk of incident kidney disease, determined as incident eGFR < 60 mL/min/1.73 m^2^, has been evaluated in a retrospective study enrolling 1,073 elderly participants. In this study, serum bicarbonate level was calculated using a blood gas analyzer from the Henderson-Hasselbalch equation. Kidney function was estimated at baseline and seven years later from values of serum creatinine and cystatin C, calculating the eGFR using the CKD-EPI (CKD Epidemiology collaboration) creatinine-cystatin C equation. No assessment of kidney function was performed in the intervening years. In adjusted models, the association between lower bicarbonate concentration and incident eGFR < 60 mL/min/1.73 m^2^ failed to reach statistical significance. Therefore, compared with participants with serum bicarbonate concentration of 23–28 mM, those with lower serum bicarbonate level were not significantly at increased risk for incident eGFR < 60 mL/min/1.73 m^2^. In addition, compared to participants with bicarbonate values in the 23 to 28 mM category, those with lower bicarbonate levels did not have significant odds of rapid kidney function decline [71].

The association between serum bicarbonate concentration and change in kidney function was retrospectively examined in 5,810 participants in the MESA (Multi-Ethnic Study of Atherosclerosis) cohort, which was originally designed to assess subclinical cardiovascular disease. Total serum carbon dioxide was measured at baseline in long-term stored serum samples that had been shipped to the central laboratory. This value was assumed to be the serum bicarbonate concentration. Kidney function was estimated at baseline and at examinations 3 and 4 by serum creatinine and cystatin C concentration, calculating the eGFR using the CKD-EPI creatinine-cystatin C equation. The baseline eGFR was > 60 mL/min/1.73 m^2^ and the average bicarbonate concentration was 23.2 ± 1.8 mEq/L. In this study, serum bicarbonate categories were not associated significantly with adjusted risk of incident reduced eGFR. Similar findings were observed when serum bicarbonate level was analyzed as a categorical variable. Participants with serum bicarbonate concentration < 23 mEq/L compared to higher levels were not significantly associated with incident reduced eGFR (OR, 1.17; 95% CI, 0.99–1.39). The incidence rate ratio for the association of bicarbonate level < 21 mEq/L relative to 23-24 mEq/L was 1.16 (95% CI, 0.83–1.62) for incident reduced eGFR. In adjusted analysis, lower baseline bicarbonate concentration was associated with higher odds for rapid kidney function decline, but assessment of the influence of serum bicarbonate concentration on kidney function change in this study is constrained by the limited estimation of kidney function. The low average serum bicarbonate value in persons with well preserved kidney function is remarkable in this population, which perhaps may be explained by the manner in which serum bicarbonate was measured, as stored samples may lose CO_2_. Also unexpected in this study is the independent association between lower eGFR and higher bicarbonate concentration in adjusted linear regression analysis [72].

#### 2.7.5. Influence of Sodium Bicarbonate Therapy on Chronic Kidney Disease Progression

Results from recent clinical trials might suggest that correction or prevention of metabolic acidosis by alkali supplementation with sodium citrate or sodium bicarbonate may be applied to slow the decline of kidney function. However, limitations of the trials and miscellaneous therapeutic approaches prevent these studies from providing sufficient evidence to substantiate the application of this renoprotective strategy (Table 3). Further evaluation concerning potential risks of chronic bicarbonate therapy is also required [65, 66].

In a randomized single-center study including 134 adults with GFR 15 to 30 mL/min/1.73 m^2^ and serum bicarbonate level 16 to 20 mM, patients were assigned to either supplementation with oral sodium bicarbonate (12–31 mEq/day, mean 1.2 g/day) or standard care for 2 years. Compared to the control group, patients supplemented with oral sodium bicarbonate are less likely to experience rapid progression of CKD and fewer of them develop ESRD [73]. This study enrolls participants with serum bicarbonate level 16 to 20 mM, excluding most patients with CKD, as only approximately 5% of CKD patients show a serum bicarbonate level < 20 mM. Consequently, applying these results to populations of CKD patients with higher serum bicarbonate values is not straightforward [51, 68, 74].

In a study enrolling 59 patients with hypertensive nephropathy (mean eGFR 33 mL/min, range 20–60 mL/min) and metabolic acidosis (baseline serum bicarbonate level < 22 mM), 30 patients accepted treatment with sodium citrate (1 mEq/Kg bicarbonate equivalent daily) and the remaining 29 (who were unable or unwilling to take this medication) served as controls. After two years of therapy, the eGFR was higher and the rate of eGFR decline was slower in patients who received sodium citrate, compared to the control group. Plasma ionized calcium level decreased in patients treated with sodium citrate but remained constant in the control group [75].

The effect of sodium bicarbonate administration on the rate of progression of CKD has been investigated in a 5-year prospective, randomized, and controlled interventional study in patients with hypertensive nephropathy and a serum bicarbonate level of at least 24.5 mM. Participants received either placebo or equimolar sodium chloride or sodium bicarbonate. After 5 years, the eGFR using plasma cystatin C concentrations is higher and the rate of cystatin C-eGFR decline is slower in patients given sodium bicarbonate than in those given placebo or sodium chloride. However, this reduction in the rate of progression among patients included in the sodium bicarbonate group is not observed using other estimates of kidney function [76].

The efficacy of fruits and vegetables ingestion has been found to be similar to that of oral sodium bicarbonate supplementation to diminish kidney injury in patients with hypertensive nephropathy at stage 1 or 2 CKD. Potential renal acid load decreases in CKD patients given fruits and vegetables while it is not modified in sodium bicarbonate-treated patients [77].

Therefore, evidence that treatment with sodium bicarbonate could slow the progression of CKD is not definitive. At the present time, three randomized controlled trials are investigating the effect of sodium bicarbonate on renal function and mortality among patients with CKD. The results from these trials are expected to offer solid evidence on the use of sodium bicarbonate as renoprotective intervention [78–80].

#### 2.7.6. Metabolic Acidosis in Hemodialysis Patients

Sodium bicarbonate is the main buffer used during maintenance hemodialysis and patients subjected to this procedure receive high doses of sodium bicarbonate for their life span. In hemodialysis patients, hyperchloremia and unmeasured anions have been found to have a similar acidifying effect, accounting for almost 90% of the metabolic acidosis cases [81]. Descending the concentration of chloride in the dialysate improves metabolic acidosis despite the fact that the dialysate level of bicarbonate remains unmodified [82]. The dialysate bicarbonate concentration has a major impact on the postdialysis serum bicarbonate level, but it does not correlate strongly with the predialysis serum bicarbonate concentration, which depends predominantly on other factors, such as protein intake and residual kidney function [83]. Higher animal protein intake is associated with a higher dietary acid generation that in turn is associated with lower predialysis bicarbonate levels [64, 84]. There is an inverse relationship between serum tCO_2_ level [85, 86] or plasma bicarbonate concentration [87, 88] and normalized protein catabolic rate, suggesting that more acidotic patients have a greater protein intake.

A number of studies have documented a U-shaped association between serum predialysis bicarbonate concentration and mortality rate among patients undergoing chronic hemodialysis, suggesting that overdosing alkali therapy may be hazardous [65]. Predialysis bicarbonate levels from 17.5 to 22.5 mM have been associated with the slightest relative risk of death. Either bicarbonate value > 22.5 mM or < 17.5 mM raises the relative risk of death [86, 89, 90]. In 2011, a retrospective case-control study conducted at Fresenius Medical Care North America facilities reported that alkalosis is a significant risk factor associated with cardiopulmonary arrest [91]. In 2012, the FDA issued a safety communication informing that predialysis serum bicarbonate level ≥ 27 mM is associated with a higher risk of cardiac arrhythmia, cardiopulmonary arrest, and death [92].

The effect of dialysate bicarbonate concentration on mortality has been assessed using data from the Dialysis Outcomes and Practice Patterns Study (DOPPS). In adjusted models, higher dialysate bicarbonate concentration is associated positively with all-cause mortality (adjusted hazard ratio 1.08 per 4 mM). There is higher mortality risk in patients treated with higher dialysate bicarbonate concentration [83].

## 3. Side Effects of Sodium Bicarbonate Therapy

A recent retrospective review of symptomatic cases involving ingestion of baking soda powder (household sodium bicarbonate) reports that 192 cases were notified to the California Poison Control System between the years 2000 and 2012 and concludes that misuse of baking soda may result in severe acid-base and electrolyte alterations or respiratory depression, particularly in children, pregnant women, alcoholics, and patients on diuretics and when baking soda is used regularly as an antacid or to alter urine drugs screen tests [93]. Therapy with sodium bicarbonate may be associated with some side effects, such as hypokalemia, ionized hypocalcemia, hypercapnia, hemodynamic instability, particularly during hemodialysis sessions, prolongation of the QTc interval, a rise in the urinary excretion of sodium, and the potential to deteriorate vascular calcifications on chronic administration (Table 4). In addition, other untoward effects of sodium bicarbonate have been inconsistently reported and they have uncertain clinical significance.

### 3.1. Hypokalemia

A reduction in the serum potassium level has been observed following sodium bicarbonate administration in patients with septic shock [38] and stage 5 CKD [94]. During DKA management, an increase in the amount of potassium required to maintain a normal plasma value has been detected in patients treated with sodium bicarbonate by some [30, 95] but not all [24] studies.

### 3.2. Ionized Hypocalcemia

Sodium bicarbonate infusion reduces plasma ionized calcium concentration in critically ill patients with metabolic acidosis [21, 38]. In vitro, bicarbonate concentration has a major effect reducing ionized calcium level in serum [96]. It has been suggested that the drop in the plasma ionized calcium concentration may contribute to decrease in cardiac and vascular contractility and responsiveness to catecholamines [40].

### 3.3. Hypercapnia

The infusion of sodium bicarbonate has been consistently associated with an increase in carbon dioxide production and a rise in arterial pCO_2_ [14, 21, 22, 37, 40, 41, 97–101]. Excess carbon dioxide is normally cleared by pulmonary hyperventilation, but when sodium bicarbonate is administered to patients on mechanical ventilation, adjustments have to be made to remove the excess carbon dioxide [37, 40, 98, 100]. During hemodialysis using bicarbonate buffer, there is a rapid flux of bicarbonate from the dialysate to the patient which generates excess carbon dioxide and requires an increase in ventilation to maintain acid-base balance. At a bicarbonate concentration in the dialysis fluid of 35 mM, there is a sustained rise in minute ventilation and an increase in carbon dioxide elimination [102, 103]. In patients with chronic obstructive pulmonary disease undergoing hemodialysis with dialysate bicarbonate concentration of 37 mM, the increase in arterial pCO_2_ during hemodialysis is greater than in control subjects, although both experience a rise in pCO_2_ [104].

### 3.4. Hemodynamic Instability during Hemodialysis

In a randomized crossover study, dialysate bicarbonate level of 32 mM is associated with lower blood pressure and more intradialysis hypotensive episodes, as compared to bicarbonate concentration of 26 mM [84]. Similar results have been documented comparing dialysate bicarbonate concentration 40 mM versus 30 mM [105]. As mentioned, according to a safety communication issued by the FDA in 2012, predialysis serum bicarbonate level ≥ 27 mM is associated with a higher risk of low blood pressure, hypokalemia, hypoxemia, hypercapnia, cardiac arrhythmia, cardiopulmonary arrest, and death [92].

### 3.5. Prolongation of the Corrected QT (QTc) Interval

A randomized controlled crossover trial has found that the QTc interval is prolonged when the dialysis fluid contains high bicarbonate, low potassium, and low ionized calcium concentrations. High bicarbonate concentration in dialysate per se is an independent predictor of the QTc interval, such that prolongation in the QTc interval occurs more frequently with dialysate containing high bicarbonate concentration whatever the level of ionized calcium and potassium. The QTc interval prolongation in hemodialysis patients persists long after the end of the dialysis session. Prolonged QTc interval has been held responsible for cardiovascular mortality both in healthy subjects and in uremic patients [106].

### 3.6. Increased Urinary Sodium Excretion

An increase in the urinary excretion of sodium has been consistently observed following sodium bicarbonate supplementation to patients with several stages of CKD [73, 77, 94].

### 3.7. Progression of Vascular Calcifications

Vascular calcification, caused by the deposition of calcium salt crystals (predominantly hydroxyapatite) in the arterial wall, is extremely common among patients with CKD and shows a marked tendency to progress among patients undergoing dialysis, in which survival is inversely related to the extent of vascular calcification. The degree of arterial calcification has been positively correlated with older age, male sex, white race, diabetes, daily dose of calcium-containing phosphate binders, duration of hypertension, and longer periods on dialysis, but these risk factors explain only partially the appearance and progression of vascular calcification [107].

Patients with CKD are frequently supplemented with carbonate and bicarbonate anions and patients on hemodialysis suffer periodic bicarbonate loading during the procedure. The potential effect of this long-life high-dose carbonate and bicarbonate supplementation on vascular calcification among patients with CKD, particularly undergoing hemodialysis, has been barely examined.

It has been long known that sodium bicarbonate and calcium mixed together in the same solution may form an insoluble precipitate, calcium carbonate (CaCO_3_), although the mechanism of precipitation is poorly understood. Calcium carbonate has been observed to precipitate in the fluid pathway of dialysate delivery systems, partially occluding the fluid pathway and leading to system malfunction [108]. Serum calcium may bind to carbonate and bicarbonate anions resulting in the formation of calcium carbonate and calcium bicarbonate (CaHCO_3_^+^), respectively [96, 109, 110]. Under physiological conditions, only a minor fraction of the calcium in serum is bound to bicarbonate, having been estimated that calcium bicarbonate represents 3% of the total calcium in blood [109]. The amount of calcium carbonate or calcium bicarbonate complexes that can be generated in plasma when either of these anions is supplemented and their potential role causing or worsening vascular calcifications have not been investigated.

Bicarbonate metabolism is linked to the activity of isoenzymes of carbonic anhydrase, which in turn have been recently involved in physiological bone calcification, as carbonic anhydrase action is crucial to promote calcium carbonate precipitation. Calcium carbonate deposits are only 10% of bone tissue, but they play an essential role in the formation of the calcium phosphate (hydroxyapatite) crystal, by acting as a calcium core that precedes calcium phosphate deposition. In vitro experiments suggest that carbonic anhydrase may play an essential role in new bone formation, by inducing formation of calcium carbonate and subsequent calcification under physiological conditions. Following treatment with acetazolamide, calcium nodule formation was decreased in cultured cells [111, 112].

In addition, carbonic anhydrase isoenzymes may be important to activate matrix Gla protein, a regulator of vascular calcification produced by vascular smooth muscle cells. To become active, this protein requires *γ*-carboxylation of glutamate residues, a vitamin K-dependent process that also necessitates a supply of carbon dioxide or bicarbonate, which may involve carbonic anhydrase action. Whether carbonic anhydrase isoforms play a role in vascular calcification in patients with CKD has not been explored.

### 3.8. Side Effects of Sodium Bicarbonate with Uncertain Clinical Significance

Side effects of sodium bicarbonate therapy inconsistently found or with unclear clinical consequences include impairment of tissue oxygenation, intracellular acidosis, paradoxical cerebrospinal fluid acidosis, hyperosmolar state, increased lactate production, and slight blood pressure reduction.

Hemoglobin binds oxygen in the lungs as carbon dioxide is excreted and releases oxygen in the peripheral tissues, where the affinity of hemoglobin for oxygen falls due to high CO_2_ concentrations and low pH. The increase of 2,3-bisphosphoglycerate inside the red cells contributes to release of oxygen to the tissues by binding to deoxyhemoglobin. It has been suggested that bicarbonate administration may impair tissue oxygenation by increasing the affinity of hemoglobin for oxygen, particularly in patients with diabetic ketoacidosis in whom the concentration of 2,3-bisphosphoglycerate is reduced inside the erythrocytes. In these patients, tissue acidosis helps to maintain tissue oxygenation by enhancing oxygen delivery from erythrocytes. Administration of bicarbonate reduces acidosis and might be followed by tissue hypoxia [25, 113]. However, impairment in tissue oxygenation has not been found following sodium bicarbonate therapy [22, 114]. In vitro studies using human platelets [115] and leucocytes [116] have documented intracellular acidification following bicarbonate addition to the medium. In healthy volunteers, a decrease in the intracellular pH of the brain (calculated from the chemical shift of inorganic phosphate measured by magnetic resonance spectroscopy) has been found after bicarbonate infusion [101]. Paradoxical worsening of the cerebrospinal fluid acidosis has been inconsistently found as a result of sodium bicarbonate administration [6, 23, 113]. A hyperosmolal state has been documented in an early study in patients after cardiac resuscitation [117]. In some studies, sodium bicarbonate therapy has been associated with an increase in lactate production and a rise in the blood lactate concentration [18, 26, 98, 118], but this effect has not been found in other investigations [22, 27]. A slight reduction in systolic blood pressure has been an inconsistent finding associated with sodium bicarbonate administration [77, 119].

## 4. Conclusive Remarks

Metabolic acidosis is a common acid-base disorder and its management should be directed by the current guidelines of therapy. The utility of sodium bicarbonate replacement in conditions associated with loss of sodium bicarbonate is widely accepted, such as renal proximal tubular acidosis and diarrhea, for in these disorders the loss of sodium bicarbonate contributes to generation of the acidosis. However, evidence of more favorable clinical outcomes associated with the use of symptomatic therapy with sodium bicarbonate in order to increase plasma pH in most acute conditions featuring metabolic acidosis is not definitive in humans. Available evidence suggests that the severity of metabolic acidosis in these conditions reflects the gravity of the underlying illness rather than being itself a contributor to mortality. Sodium bicarbonate administration usually, although not always, corrects the acidosis, rising serum bicarbonate concentration, serum pH, and the partial pressure of carbon dioxide, but evidence for clinical benefit derived from this effect is not conclusive. Therapy of such situations should be focused on the cause of the acidosis. On the other hand, management of chronic conditions leading to metabolic acidosis, such as kidney disease, may be challenging. Nonetheless, evidence for a net beneficial effect of symptomatic sodium bicarbonate administration to correct the acidosis in this condition is not compelling and further studies are needed to establish its therapeutic value [13, 37, 40, 64]. Recent studies have suggested that metabolic acidosis might contribute to worsening kidney disease and sodium bicarbonate supplementation has been proposed as a renoprotective strategy. However, limitations of these studies prevent reaching definite conclusions and further investigations are required in order to ensure the validity of this therapeutic approach [79].

## Figures and Tables

**Table 1:** Acute conditions in which sodium bicarbonate therapy has not improved outcomes.

Acute conditions in which sodium bicarbonate therapy does not improve outcomes	
Diabetic ketoacidosis	
Lactic acidosis	
Septic shock	
Cardiac arrest	
Intraoperative metabolic acidosis	

**Table 2:** Relationship between metabolic acidosis and kidney disease progression.

Type of study	Patients number	Main finding	Limitations	Reference
Retrospective	5,422	Weak association between low serum bicarbonate and progression of kidney disease	Only 9% of the participants have estimated glomerular filtration rate (eGFR) <60 mL/min/1.73 m^2^ at baseline The estimation of progression of kidney disease is very limited	Shah et al., 2009 [67]
Retrospective	1,094	Higher serum bicarbonate is associated with reduced hazard of kidney disease progression	It is a secondary analysis Limited external validity	Raphael et al., 2011 [68]
Prospective, multicenter cohort	3,939	The risk of disease progression is 3% lower per 1 mM increase in serum bicarbonate level	Hazard ratio 0.97, 95% confidence interval 0.94–0.99	Dobre et al., 2013 [51]
Retrospective	113	Lower bicarbonate level is associated with high risk of kidney disease progression	At baseline, patients in the low-bicarbonate group have strikingly more impaired kidney function compared to patients in the high-bicarbonate group Small sample size High number of censored observations	Kanda et al., 2013 [70]
Retrospective	1,073	There is no significant association between lower bicarbonate level and incident eGFR <60 mL/min/1.73 m^2^	Serum bicarbonate level was calculated from the Henderson-Hasselbalch equation	Goldenstein et al., 2014 [71]
Retrospective	5,810	Serum bicarbonate categories are not associated with adjusted risk of incident eGFR <60 mL/min/1.73 m^2^	Total serum carbon dioxide was measured at baseline in serum samples long-term stored	Driver et al., 2014 [72]
Retrospective	632	No clear association between higher quartiles of net endogenous acid production (NEAP) and faster decline in GFR over follow-up	Higher quartiles of NEAP are associated with a faster decline in GFR, but there is no association in time-to-event analyses	Scialla et al., 2012 [69]

**Table 3:** Influence of sodium bicarbonate therapy on chronic kidney disease progression.

Type of study	Patients number	Main finding	Limitations	Reference
Randomized, single center	134	Patients supplemented with oral sodium bicarbonate are less likely to experience rapid progression of kidney disease.	Serum bicarbonate level in participants was 16 to 20 mM, limiting external validity.	de Brito-Ashurst et al., 2009 [73]
Prospective	59	The rate of estimated glomerular filtration rate (eGFR) decline was slower in patients who received sodium citrate.	The control group was composed of patients that were unable to take the medication.	Phisitkul et al., 2010 [75]
Prospective, randomized, controlled	120	The rate of cystatin C-eGFR decline is slower in patients given sodium bicarbonate.	The reduction in the rate of progression is not observed using other estimates of kidney function.	Mahajan et al., 2010 [76]

**Table 4:** Side effects of sodium bicarbonate therapy.

Side effects of sodium bicarbonate therapy	
Hypokalemia	
Ionized hypocalcemia	
Prolongation of the QTc interval	
Hypercapnia	
Hemodynamic instability during hemodialysis	
Increase in urinary sodium excretion	
Potential progression of vascular calcifications	
Side effects of sodium bicarbonate with uncertain clinical significance	
(i) Impairment of tissue oxygenation	
(ii) Intracellular acidosis	
(iii) Paradoxical cerebrospinal fluid acidosis	
(iv) Hyperosmolar state	
(v) Increased lactate production	
(vi) Slight blood pressure reduction	
